# 2,2′-(1,3-Diazinane-1,3-di­yl)diaceto­nitrile: a second monoclinic polymorph

**DOI:** 10.1107/S1600536811038013

**Published:** 2011-09-30

**Authors:** Augusto Rivera, Mauricio Maldonado, Jaime Ríos-Motta, Karla Fejfarová, Michal Dušek

**Affiliations:** a Departamento de Química, Universidad Nacional de Colombia, Ciudad Universitaria, Bogotá, Colombia; b Institute of Physics ASCR, v.v.i., Na Slovance 2, 182 21 Praha 8, Czech Republic

## Abstract

A new monoclinic polymorph of the title compound, C_8_H_12_N_4_, in the space group *P*2*_1_/n* (*Z* = 4) is reported. The previously known form was also monoclinic, *P*2_1_ */c* (*Z* = 4), but the unit-cell parameters and crystal packing were different [Shoja & Saba (1993 ▶). *Acta Cryst.* C**49**, 354–355]. The hexa­hydro­pyrimidine ring of the title compound adopts a chair conformation with a diequatorial substitution and with the CH_2_-C N groups oriented nearly parallel and in the same direction [NC—CH_2_⋯CH_2_—CN pseudo torsion angle = −6.27 (18)°]. In the crystal, inter­molecular C—H⋯ N hydrogen bonds connects the mol­ecules into a chain along the *b* axis.

## Related literature

For the original monoclinic polymorph, see: Shoja & Saba (1993 ▶). For the synthesis of the title compound, see: Rivera *et al.* (2004 ▶); Katritzky *et al.* (1990 ▶). For the use of nitriles in synthesis, see: Prasad & Bhalla (2010 ▶).
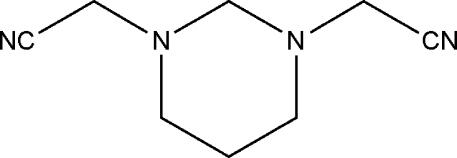


## Experimental

### 

#### Crystal data

C_8_H_12_N_4_
*M* *_r_* = 164.2
Monoclinic, 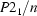

*a* = 11.1300 (6) Å
*b* = 6.3501 (3) Å
*c* = 13.1373 (7) Å
β = 102.066 (6)°
*V* = 907.99 (8) Å^3^
*Z* = 4
Cu *K*α radiation
μ = 0.63 mm^−1^
*T* = 120 K
0.16 × 0.09 × 0.01 mm

#### Data collection

Agilent Gemini Ultra diffractometer with an Atlas CCD detector
Absorption correction: multi-scan (*CrysAlis PRO*; Agilent, 2010 ▶) *T*_min_ = 0.864, *T*_max_ = 1.000
2863 measured reflections
1404 independent reflections
929 reflections with *I* > 3σ(*I*)
*R*_int_ = 0.027

#### Refinement

*R*[*F* ^2^ > 2σ(*F* ^2^)] = 0.039
*wR*(*F* ^2^) = 0.093
*S* = 1.30
1404 reflections
109 parameters
H-atom parameters constrained
Δρ_max_ = 0.18 e Å^−3^
Δρ_min_ = −0.18 e Å^−3^

### 

Data collection: *CrysAlis PRO* (Agilent, 2010 ▶); cell refinement: *CrysAlis PRO*; data reduction: *CrysAlis PRO*; program(s) used to solve structure: *SIR2002* (Burla *et al.*, 2003 ▶); program(s) used to refine structure: *JANA2006* (Petříček *et al.* 2006 ▶); molecular graphics: *DIAMOND* (Brandenburg & Putz, 2005 ▶); software used to prepare material for publication: *JANA2006*.

## Supplementary Material

Crystal structure: contains datablock(s) global, I. DOI: 10.1107/S1600536811038013/gk2401sup1.cif

Structure factors: contains datablock(s) I. DOI: 10.1107/S1600536811038013/gk2401Isup2.hkl

Supplementary material file. DOI: 10.1107/S1600536811038013/gk2401Isup3.cml

Additional supplementary materials:  crystallographic information; 3D view; checkCIF report

## Figures and Tables

**Table 1:** Hydrogen-bond geometry (Å, °)

*D*—H⋯*A*	*D*—H	H⋯*A*	*D*⋯*A*	*D*—H⋯*A*
C7—H7B⋯N4^i^	0.96	2.59	3.396 (3)	141

Symmetry code: (i) 
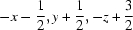
.

**Fig. 1.**
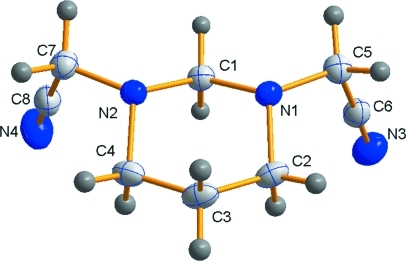
A view of the title molecule. Displacement ellipsoids are drawn at the 50% probability level.

**Fig. 2.**
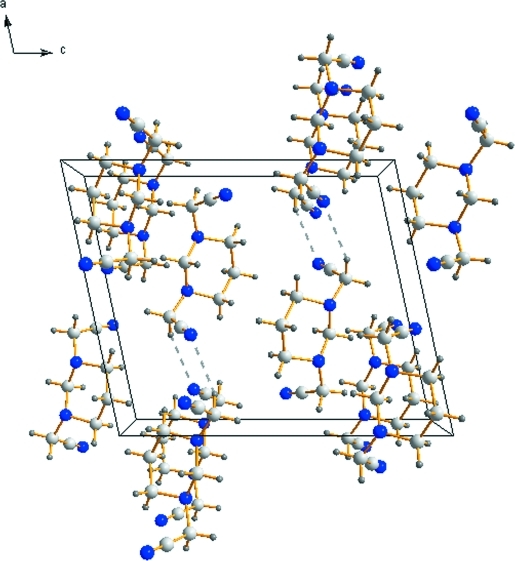
Packing of the molecules of the title compound view along the b axis. Dashed lines represent intermolecular hydrogen bonds.

## supplementary crystallographic information

### Comment

Nitriles are widely used starting materials and intermediates in organic synthesis. For instance, hydration of nitriles to corresponding carboxamides is an important reaction in nature and organic synthesis (Prasad & Bhalla, 2010). The title compound (I) was synthesized by one-step reaction between the macrocyclic aminal 1,3,7,9,13,15,19,21-octaazapentacyclo- [19.3.1.1^3,7^.1^9,13^.1^15,19^]octacosane and hydrocyanic acid according to a methodology previously published (Rivera, *et al.* 2004). Single crystals of title compound were obtained by recrystallization from EtOH solution. An alternative synthetic method for the preparation of title compound involves the substitution of benzotriazolyl groups by cyano anion from the key benzotriazolyl intermediate 1,1'-(dihydropyrimidine-1,3(2*H*,4*H*)- diyldimethanediyl)-bis(1*H*-benzotriazole) (Katritzky *et al.*, 1990)

The molecular structure and atom-numbering scheme for (I) are shown in Fig. 1. The cell dimensions of the title modification differ from the cell dimensions of the previously reported monoclinic P2*_1_/c* modification: a = 8.303, b = 8.733, c = 12.998 Å; β = 107.73°, V = 897.7 Å^3^ (Shoja & Saba 1993). The bond lengths and angles are comparable with the previously reported polymorph.

In the crystal packing (Fig. 2) C—H··· N hydrogen bonds connect the molecules into a chain along the *b* axis. The C···N distances [3.396 (3) Å] is significantly shorter than the corresponding C···N distances in previously reported polymorph [3.518 (6) Å] (Shoja & Saba 1993), but the N···H distance in the title compound [2.59 Å] is longer than the corresponding N···H in mentionated polymorph [2.547 (18) Å].

### Experimental

For the originally reported synthesis, see: Rivera *et al.* (2004). Single crystals of the title compound were obtained by recrystallization from EtOH solution (m.p. 340 K).

### Refinement

Hydrogen atoms were added in calculated positions and refined as riding with C–H distance of 0.96 Å The isotropic atomic displacement parameters of hydrogen atoms were evaluated as 1.2×*U*_eq_ of the parent atom.

### Crystal data

**Table:**

C_8_H_12_N_4_	*F*(000) = 352
*M**_r_* = 164.2	*D*_x_ = 1.201 Mg m^−^^3^
Monoclinic, *P*2_1_/*n*	Cu *K*α radiation, λ = 1.5418 Å
Hall symbol: -P 2yn	Cell parameters from 1334 reflections
*a* = 11.1300 (6) Å	θ = 3.4–64.4°
*b* = 6.3501 (3) Å	µ = 0.63 mm^−^^1^
*c* = 13.1373 (7) Å	*T* = 120 K
β = 102.066 (6)°	Plate, colourless
*V* = 907.99 (8) Å^3^	0.16 × 0.09 × 0.01 mm
*Z* = 4	

### Data collection

**Table:**

Agilent Gemini Ultra diffractometer with an Atlas CCD detector	1404 independent reflections
Radiation source: Enhance Ultra (Cu) X-ray Source	929 reflections with *I* > 3σ(*I*)
mirror	*R*_int_ = 0.027
Detector resolution: 10.3784 pixels mm^-1^	θ_max_ = 64.6°, θ_min_ = 4.8°
Rotation method data acquisition using ω scans	*h* = −11→12
Absorption correction: multi-scan (*CrysAlis PRO*; Agilent, 2010)	*k* = −7→3
*T*_min_ = 0.864, *T*_max_ = 1.000	*l* = −14→14
2863 measured reflections	

### Refinement

**Table:**

Refinement on *F*^2^	48 constraints
*R*[*F*^2^ > 2σ(*F*^2^)] = 0.039	H-atom parameters constrained
*wR*(*F*^2^) = 0.093	Weighting scheme based on measured s.u.'s *w* = 1/[σ^2^(*I*) + 0.0009*I*^2^]
*S* = 1.30	(Δ/σ)_max_ = 0.001
1404 reflections	Δρ_max_ = 0.18 e Å^−^^3^
109 parameters	Δρ_min_ = −0.18 e Å^−^^3^
0 restraints	

### Special details

**Table:**

Refinement. The refinement was carried out against all reflections. The conventional *R*-factor is always based on *F*. The goodness of fit as well as the weighted *R*-factor are based on *F* and *F*^2^ for refinement carried out on *F* and *F*^2^, respectively. The threshold expression is used only for calculating *R*-factors *etc*. and it is not relevant to the choice of reflections for refinement.The program used for refinement, Jana2006, uses the weighting scheme based on the experimental expectations, see _refine_ls_weighting_details, that does not force *S* to be one. Therefore the values of *S* are usually larger than the ones from the *SHELX* program.

### Fractional atomic coordinates and isotropic or equivalent isotropic displacement parameters (Å^2^)

**Table:**

	*x*	*y*	*z*	*U*_iso_*/*U*_eq_	
N1	0.24013 (14)	0.1540 (2)	0.84921 (11)	0.0221 (5)	
N2	0.02686 (14)	0.1567 (2)	0.77670 (12)	0.0227 (5)	
N3	0.38363 (17)	−0.2911 (3)	0.97150 (14)	0.0368 (7)	
N4	−0.13683 (17)	−0.3109 (3)	0.77144 (15)	0.0432 (7)	
C1	0.13764 (16)	0.0291 (3)	0.79354 (14)	0.0219 (6)	
C2	0.22087 (19)	0.2149 (3)	0.95257 (15)	0.0283 (7)	
C3	0.10159 (19)	0.3355 (3)	0.94117 (15)	0.0314 (7)	
C4	−0.00483 (19)	0.2148 (3)	0.87606 (15)	0.0294 (7)	
C5	0.35671 (17)	0.0485 (3)	0.85421 (15)	0.0266 (7)	
C6	0.37333 (18)	−0.1454 (3)	0.91936 (16)	0.0268 (7)	
C7	−0.07465 (18)	0.0560 (3)	0.70529 (16)	0.0276 (7)	
C8	−0.11174 (18)	−0.1512 (4)	0.74169 (16)	0.0310 (7)	
H1a	0.153761	−0.013785	0.727602	0.0263*	
H1b	0.126995	−0.092437	0.834233	0.0263*	
H2a	0.287763	0.302129	0.98659	0.034*	
H2b	0.217385	0.090842	0.993595	0.034*	
H3a	0.109451	0.46939	0.909275	0.0377*	
H3b	0.084982	0.362397	1.008806	0.0377*	
H4a	−0.019698	0.089559	0.912481	0.0353*	
H4b	−0.076841	0.30205	0.86304	0.0353*	
H5a	0.422653	0.144787	0.879563	0.0319*	
H5b	0.365998	0.014494	0.785061	0.0319*	
H7a	−0.053712	0.039324	0.638472	0.0331*	
H7b	−0.144222	0.148886	0.692234	0.0331*	

### Atomic displacement parameters (Å^2^)

**Table:**

	*U*^11^	*U*^22^	*U*^33^	*U*^12^	*U*^13^	*U*^23^
N1	0.0246 (9)	0.0204 (9)	0.0211 (9)	−0.0003 (8)	0.0044 (7)	−0.0017 (7)
N2	0.0234 (9)	0.0233 (9)	0.0218 (9)	0.0035 (8)	0.0057 (7)	0.0005 (7)
N3	0.0429 (12)	0.0326 (11)	0.0332 (10)	0.0053 (9)	0.0042 (9)	0.0033 (9)
N4	0.0343 (11)	0.0396 (12)	0.0549 (13)	−0.0049 (10)	0.0077 (9)	0.0104 (10)
C1	0.0248 (11)	0.0193 (10)	0.0219 (11)	0.0011 (9)	0.0053 (8)	0.0011 (8)
C2	0.0384 (12)	0.0257 (11)	0.0197 (11)	−0.0001 (10)	0.0034 (9)	−0.0023 (9)
C3	0.0442 (13)	0.0262 (11)	0.0248 (11)	0.0079 (11)	0.0093 (9)	−0.0017 (9)
C4	0.0343 (12)	0.0300 (12)	0.0269 (11)	0.0078 (10)	0.0130 (9)	0.0039 (9)
C5	0.0266 (12)	0.0246 (11)	0.0287 (11)	−0.0008 (9)	0.0058 (9)	0.0017 (9)
C6	0.0250 (11)	0.0283 (12)	0.0261 (11)	0.0024 (10)	0.0029 (9)	−0.0041 (10)
C7	0.0270 (11)	0.0280 (11)	0.0268 (11)	−0.0002 (10)	0.0033 (9)	0.0045 (9)
C8	0.0228 (11)	0.0374 (13)	0.0318 (12)	−0.0002 (11)	0.0037 (9)	0.0034 (11)

### Geometric parameters (Å, °)

**Table:**

N1—C1	1.455 (2)	C2—H2b	0.96
N1—C2	1.470 (3)	C3—C4	1.516 (3)
N1—C5	1.450 (2)	C3—H3a	0.96
N2—C1	1.453 (2)	C3—H3b	0.96
N2—C4	1.469 (3)	C4—H4a	0.96
N2—C7	1.457 (2)	C4—H4b	0.96
N3—C6	1.143 (3)	C5—C6	1.489 (3)
N4—C8	1.142 (3)	C5—H5a	0.96
C1—H1a	0.96	C5—H5b	0.96
C1—H1b	0.96	C7—C8	1.487 (3)
C2—C3	1.513 (3)	C7—H7a	0.96
C2—H2a	0.96	C7—H7b	0.96
			
C1—N1—C2	110.95 (15)	C4—C3—H3b	109.4713
C1—N1—C5	111.79 (14)	H3a—C3—H3b	107.3996
C2—N1—C5	112.44 (14)	N2—C4—C3	108.91 (17)
C1—N2—C4	111.01 (14)	N2—C4—H4a	109.471
C1—N2—C7	111.87 (15)	N2—C4—H4b	109.4718
C4—N2—C7	112.62 (16)	C3—C4—H4a	109.4714
N1—C1—N2	108.89 (15)	C3—C4—H4b	109.4711
N1—C1—H1a	109.4708	H4a—C4—H4b	110.0256
N1—C1—H1b	109.4715	N1—C5—C6	114.25 (17)
N2—C1—H1a	109.4709	N1—C5—H5a	109.4711
N2—C1—H1b	109.472	N1—C5—H5b	109.4716
H1a—C1—H1b	110.0468	C6—C5—H5a	109.4711
N1—C2—C3	109.65 (15)	C6—C5—H5b	109.4712
N1—C2—H2a	109.4713	H5a—C5—H5b	104.2272
N1—C2—H2b	109.4713	N3—C6—C5	177.6 (2)
C3—C2—H2a	109.4712	N2—C7—C8	114.28 (16)
C3—C2—H2b	109.4706	N2—C7—H7a	109.4703
H2a—C2—H2b	109.2966	N2—C7—H7b	109.4712
C2—C3—C4	111.47 (17)	C8—C7—H7a	109.4713
C2—C3—H3a	109.4714	C8—C7—H7b	109.4717
C2—C3—H3b	109.4712	H7a—C7—H7b	104.193
C4—C3—H3a	109.471	N4—C8—C7	178.0 (2)

### Hydrogen-bond geometry (Å, °)

**Table:**

*D*—H···*A*	*D*—H	H···*A*	*D*···*A*	*D*—H···*A*
C7—H7B···N4^i^	0.96	2.59	3.396 (3)	141

Symmetry codes: (i) −*x*−1/2, *y*+1/2, −*z*+3/2.
